# Tauroursodeoxycholic acid: a bile acid that may be used for the prevention and treatment of Alzheimer’s disease

**DOI:** 10.3389/fnins.2024.1348844

**Published:** 2024-02-19

**Authors:** Honghu Song, Jiancheng Liu, Linjie Wang, Xiaomin Hu, Jiayu Li, Li Zhu, Rizhao Pang, Anren Zhang

**Affiliations:** ^1^School of Health Preservation and Rehabilitation, Chengdu University of Traditional Chinese Medicine, Chengdu, China; ^2^Department of Rehabilitation Medicine, General Hospital of Western Theater Command, Chengdu, China; ^3^Department of Rehabilitation Medicine, Shanghai Fourth People's Hospital Affiliated to Tongji University, Shanghai, China

**Keywords:** tauroursodeoxycholic acid, Alzheimer’s disease, bile acids, neurodegenerative diseases, mechanisms

## Abstract

Alzheimer’s disease (AD) is a prevalent neurodegenerative disease that has become one of the main factors affecting human health. It has serious impacts on individuals, families, and society. With the development of population aging, the incidence of AD will further increase worldwide. Emerging evidence suggests that many physiological metabolic processes, such as lipid metabolism, are implicated in the pathogenesis of AD. Bile acids, as the main undertakers of lipid metabolism, play an important role in the occurrence and development of Alzheimer’s disease. Tauroursodeoxycholic acid, an endogenous bile acid, has been proven to possess therapeutic effects in different neurodegenerative diseases, including Alzheimer’s disease. This review tries to find the relationship between bile acid metabolism and AD, as well as explore the therapeutic potential of bile acid taurocursodeoxycholic acid for this disease. The potential mechanisms of taurocursodeoxycholic acid may include reducing the deposition of Amyloid-β protein, regulating apoptotic pathways, preventing tau hyperphosphorylation and aggregation, protecting neuronal synapses, exhibiting anti-inflammatory properties, and improving metabolic disorders. The objective of this study is to shed light on the use of tauroursodeoxycholic acid preparations in the prevention and treatment of AD, with the aim of identifying effective treatment targets and clarifying various treatment mechanisms involved in this disease.

## 1 Introduction

Alzheimer’s disease (AD) is a hidden neurodegenerative disease that seriously impacts the daily lives of affected individuals. The clinical symptoms of AD, like dementia, worsen progressively and may endure throughout the course of a patient’s life. The dementia symptoms experienced by AD patients not only seriously affect their daily life, but also greatly amplify the burden on their families in terms of caregiving responsibilities (Mausbach et al., 2012; Schiffczyk et al., 2013). According to the International Alzheimer’s Association (ADI), the global population of dementia patients exceeds 50 million. As the leading cause of dementia, AD is one of the top 10 causes of death worldwide, and its prevalence continues to rise annually. However, the underlying causes of the disease remain unclear. Currently, it is widely acknowledged that Amyloid-β (Aβ) imbalance in generation and clearance, Tau pathology, as well as other factors such as metabolic impairment and calcium dyshomeostasis, are significant contributors to neuronal degeneration and dementia (Baker-Nigh et al., 2015; Calvo-Rodriguez et al., 2020; Mamun et al., 2020; Sultana et al., 2023). The etiology of AD may be the result of the interaction of multiple factors such as genetic, biological, and sociopsychological factors. APOE ε4 alleles are particularly strong genetic risk factors for AD (Troutwine et al., 2022). It has also been reported that age, dietary habits, insufficient sleep, psychological status, cardiovascular disease, brain injury, and other heterogeneous factors are associated with Alzheimer’s disease (James and Bennett, 2019). While progress has been made in understanding the multifactorial etiology of dementia, the prevalence and mortality rates of AD are still on the rise globally (Tahami Monfared et al., 2022). Especially, previous research has indicated a potential association between AD and COVID-19, with a significantly elevated risk of developing the disease following infection with COVID-19 (Wang L. et al., 2022; Li et al., 2023). Although much more work is needed to conclusively establish the relationship between COVID-19 and AD, it is crucial to acknowledge that the post pandemic era could pose serious challenges for the diagnosis and treatment of AD. Furthermore, AD also encompasses a myriad of associated issues that extend beyond the disease itself. Research has demonstrated that Alzheimer’s disease is closely linked to the occurrence and development of conditions such as diabetes (Gao et al., 2016; Zhu et al., 2020), thrombosis (Canobbio et al., 2016; Zamolodchikov et al., 2016), and depression (Andreasen et al., 2014). The combination of multiple diseases further increases the difficulty of clinical treatment for AD.

Research suggests that neurodegenerative diseases may originate from early life movements and trauma (Gardner and Yaffe, 2015; Ueda et al., 2023), gradually worsening with age (Armstrong, 2020). The middle-aged and elderly period is a period of gradual decline and loss of human tissue and organ function. Neurological dysfunction that occurs during this period can cause serious damage to quality of life and social participation. With the global prevalence of Alzheimer’s disease is expected to continue to rise as life expectancy increases and demographic aging occurs, exploring effective treatment methods for Alzheimer’s disease is becoming increasingly important. Consequently, there is a pressing need to develop novel treatment approaches to combat this medical challenge.

Currently, there is a growing body of evidence indicating a strong link between metabolic disorders and Alzheimer’s disease (St. John-Williams et al., 2019; Mejido et al., 2020; Sultana et al., 2023), metabolic disorders are considered a significant cause of AD (Poddar et al., 2021). In particular, the imbalances in lipid metabolism, insulin resistance, and the resultant conditions of obesity and diabetes are viewed as potential risk factors for AD (Robinson et al., 2017; Čater and Hölter, 2022). Hence, metabolic pathways may themselves contain promising therapeutic targets for AD (Cai et al., 2012). Given their significant involvement in the regulation of metabolic disorders (Belda et al., 2022; Shi et al., 2023) and the central nervous system (Rutsch et al., 2020), the gut microbiota and gut-brain axis offer new possibilities and directions for research in the treatment of neurodegenerative diseases such as AD (Doifode et al., 2021; Chiu and Anderton, 2023). Several mechanisms are involved in the gut-brain axis, including metabolite secretion, neural, and immune regulation, thus affecting cognitive function and the development of AD (Jiang et al., 2017; Giau et al., 2018; Liu et al., 2020; Kesika et al., 2021). Unhealthy diet and habits, which have long been identified as major contributors to metabolic disorders (Chauhan et al., 2022), also indicate a correlation between the increasing incidence of AD over years and the gut-brain axis. Bile acids (BAs) as microbe-derived neuroactive molecule, could regulate the gut-brain axis by participating in the above mechanisms (Wu et al., 2020; Huang et al., 2022; Yeo et al., 2023). Specifically, on the one hand, the gut microbiota directly affects the production of bile acids, which in turn shape the composition of the gut microbiota (Collins et al., 2023); on the other hand, the presence of BAs and their receptors in the brain implies a direct effect of BAs on the regulation of neurological functions (Kowalski and Mulak, 2019). Therefore, bile acids may play a role in neurodegenerative diseases such as AD (Wang et al., 2023). In recent years, there have been numerous studies investigating the therapeutic interventions involving bile acid-mediated treatment for neurodegenerative diseases, including cognitive impairment and Alzheimer’s disease (Grant and DeMorrow, 2020; Nie et al., 2022). Encouragingly, both experimental and clinical evidence suggests that bile acids, particularly hydrophilic ones, could act as potential disease modifiers for the treatment of neurodegenerative disorders (Khalaf et al., 2022). Furthermore, multiple studies have found that bile acids have the ability to affect brain function and regulate the occurrence and progression of Alzheimer’s disease (Mulak, 2021; Lirong et al., 2022). There is a significant difference in bile acid metabolism between individuals afflicted with Alzheimer’s disease and healthy individuals (Griffiths et al., 2019; Baloni et al., 2020). In fact, studies have also demonstrated that bile acid disorders have been observed in AD animal models (Kaur et al., 2021). Thus, targeted regulation of bile acids has become a new strategy for the prevention and treatment of Alzheimer’s disease (Lirong et al., 2022).

Tauroursodeoxycholic acid (TUDCA) is an endogenous bile acid commonly utilized in research to alleviate symptoms associated with neurodegenerative diseases. It has been proven to possess neuroprotective effects in models of Alzheimer’s disease (Zangerolamo et al., 2021b). For instance, when AD mice were fed a diet containing 0.4% TUDCA, there was a notable reduction in glial activation and loss of neuronal integrity when compared with untreated mice. Moreover, the accumulation of Aβ deposits in the brain was significantly diminished, leading to a marked improvement in memory deficits (Nunes et al., 2012).

This review focuses on AD as the subject of research, presents the connection between AD and bile acids, and elaborates on the potential mechanisms of the occurrence and development of AD, as well as the impact of TUDCA on these mechanisms, aiming to further investigate the therapeutic effects of TUDCA on AD and improve the existing understanding of the intervention of TUDCA in AD. In addition, recent advancements in AD treatment with TUDCA are summarized in Table 1. Ultimately, the review highlights the potential of TUDCA-related drug formulations as a new adjunct therapy for preventing the onset and progression of AD.

**Table 1:** Preclinical studies of AD treatment with TUDCA.

References	Experimental model	Intervention duration of intervention	Experimental groups	Important results bile acid effects
Dionísio et al. (2015)	APP/PS1 double-transgenic mice, maintained on a C57BL/6 J genetic background	500 mg/kg TUDCA or vehicle injection every 3 days at 7 months old. Duration:3 months	Wild-type control. Wild-type + TUDCA. APP/PS1 control. APP/PS1 + TUDCA	Attenuated Aβ deposition. Reduced amyloidogenic processing of APP and Aβ generation. Altered Akt/GSK3b activities and prevented tau hyperphosphorylation. Ameliorated astrocytosis and microgliosis. Reduced synaptic loss and improved memory.
Lo et al. (2013)	APP/PS1 double-transgenic mice, maintained on a C57BL/6 J genetic background	Diet containing 0.4% TUDCA (sodium salt) at 2 months old. Duration:6 months	Wild-type control. Wild-type + TUDCA. APP/PS1 control. APP/PS1 + TUDCA	Decreased expression of CTGF and γ-secretase activity. Reduced Aβ deposition and plaques. Improved Morris water maze performance, social recognition and passive avoidance.
Nunes et al. (2012)	APP/PS1 double-transgenic mice, maintained on a C57BL/6 J genetic background	Diet of standard laboratory chow supplemented with 0.4% (wt/wt) TUDCA (sodium salt) at 2 months old. Duration:6 months	Wild-type control. Wild-type + TUDCA. APP/PS1 control. APP/PS1 + TUDCA	Prevented APP processing, Aβ production and Aβ plaque accumulation. Inhibited activation of astrocytes and microglia. Prevents loss of neuronal integrity. Modulates lipid metabolism mediators like CTGF Rescues cognitive deficits.
Ochiai et al. (2021)	A7 transgenic mice (A7-Tg), maintained on a C57BL/6 J background	Standard chow or high-fat diet containing 32% fat at 3 months	ND control	Prevented the HFD-induced exacerbation of Aβ accumulation in brains. Reduced amyloid deposition in the brain. Attenuated diet-induced peripheral ER stress and improved systemic metabolic abnormalities. Improved age-related ER stress and metabolic abnormalities. Dietary restriction-like expression changes both in the peripheral tissues and brain.
			HFD control	
		①2 × 250 mg/kg TUDCA i.p. at 8 months old. Duration:30 days	HFD + TUDCA (i.p. injection)	
		②Intracerebroventricular administration of TUDCA (10 μg/day) at 7- to 8-month-old Duration: 28 days	HFD+TUDCA (i.c.v. injection)	
		③Drinking water containing TUDCA (1 mg/mL for 60 days, 3 mg/mL for 7 days, 5 mg/mL for 14 days, 6.5 mg/mL for 39 days) at 11-month-old. 2 × 250 mg/kg TUDCA i.P. at 14-month-old, 6 days per week. Duration:120 days		

			ND + TUDCA (drinking water + i.p. injection)	
Ramalho et al. (2006)	N2a neuroblastom cells, N2a cell lines with stable expression of APP695 (APPwt), Swedish mutant APP (APPswe) or the exon-9 deletion mutant PS1 (APPswe/ΔE9),	Preincubation with TUDCA. Duration:12 h	N2a control. N2a + TUDCA. Wt control. Wt + TUDCA. Swe control. Swe + TUDCA. Swe/ΔE9 control. Swe/ΔE9 + TUDCA	Reduced FAD-induced apoptosis. Modulated FAD-induced mitochondrial dependent apoptosis.
Ramalho et al. (2013)	Primary cultures of rat cortical and hippocampal neurons from 17- to 18-day-old fetuses of Wistar rats APP/PS1 double-transgenic mice, maintained on a C57BL/6 J genetic background	Incubated with active fragment 25 μM Aβ25-35, and 100 μM TUDCA. Duration:2–24 h. Diet of standard laboratory chow supplemented with 0.4% (wt/wt) TUDCA (sodium salt) at 2 months old. Duration:6 months	Aβ25-35 control. Aβ25-35 + TUDCA. Wild-type control. Wild-type + TUDCA. APP/PS1 control. APP/PS1 + TUDCA	Protected neurons and astrocytes from Aβ-induced toxicity. Inhibited the decrease in PSD-95. Modulated synaptic density. Prevented alterations in spontaneous synaptic activity and restored the frequency of mEPSCs.
Zangerolamo et al. (2021c)	C57BL/6 mice	STZ (3 mg/kg, ICV) was injected bilaterally into the lateral ventricles to produce the ICV-STZ AD mice model at 2-month-old. The injection was repeated 2 days after the first streptozotocin injection (1.5 mg/kg per day of injection). 300 mg/kg TUDCA i.P. at 6 days after the first application of STZ. Duration:10 days	Control Stz. Stz + TUDCA	Normalized pathological neuromarkers. Improved glucose metabolism and increases glucose-stimulated insulin secretion. Increased β-cell number per islet and total β-cell area.
Zangerolamo et al. (2021a)	C57BL/6 mice	Streptozotocin (3 mg/kg) was injected bilaterally into the lateral ventricles to generate the streptozotocin-induced Alzheimer’s disease mice model (Stz) at 2-month-old. The injection was repeated 2 days after the first streptozotocin injection (1.5 mg/kg per day of injection). 300 mg/kg TUDCA i.P. at 6 days after the first application of STZ. Duration:10 days	Control Stz. Stz + TUDCA	Lower body weight, fat pads depots and food intake. Higher RQ and EE. Reduced proinflammatory markers and orexigenic neuropeptides in the hypothalamus. Ameliorated hypothalamic leptin signaling.

Aβ, Amyloid-β; APP/PS1, amyloid precursor protein and amyloid precursor protein; CTGF, connective tissue growth factor; EE, energy expenditure; ER stress, endoplasmic reticulum stress; FAD, familial Alzheimer’s disease; HFD, high-fat diet; ICV, Intracerebroventricular; mEPSCs, miniature excitatory postsynaptic currents; ND, normal diet; PSD-95, postsynaptic density-95; RQ, Respiratory quotient; STZ, Streptozotocin; TUDCA, Tauroursodeoxycholic acid.

## 2 Alzheimer’s disease and bile acid

Bile acids are essential constituents of bile and key regulators of systemic metabolism (Ma and Patti, 2014). Maintaining normal bile acid metabolism is crucial for preventing diseases like non-alcoholic fatty liver and obesity (Perino et al., 2021). Meanwhile, it is now clear that bile acids influence a wide range of physiological processes and play an important role in the progression of many neurodegenerative diseases (Hurley et al., 2022). Notably, there is a significant link between AD and disorder of bile acid metabolism. A previous study revealed a decrease in the abundance of 7α,25-dihydroxy-3-oxocholest-4-en-26-oic acid, which is a precursor to bile acids, in the cerebrospinal fluid of patients diagnosed with AD (Griffiths et al., 2019). Another study has documented an alteration in the pathway of bile acid synthesis among patients with AD. This alteration is characterized by an increased proportion of primary bile acids, such as TCA, as well as secondary bile acids including DCA, LCA, TDCA, and GDCA. AD patients also exhibited elevated serum levels of taurine (Baloni et al., 2020), a compound that can bind to primary bile acids (Chiang, 2013) and play a neuroprotective role in the brain. The alterations in serum taurine levels suggest possible impairment of taurine transport across the blood–brain barrier (BBB) in Alzheimer’s disease (AD). Although the underlying mechanism regarding the changes in bile acid metabolism in patients with AD is currently unknown, and the specific contribution of various bile acids to the progression of AD warrants further investigation, it is evident that the disease leads to a disturbance in bile acid metabolism.

Early screening and monitoring are crucial in detecting Alzheimer’s disease, which often goes undetected in its initial stages (Zhang et al., 2021). Given the close relationship between bile acids and AD, the former may prove to be a useful biomarker for tracking the progression of the latter (Greenberg et al., 2009). Research has shown that deoxycholic acid has potential diagnostic utility in the initial phases of disease and can be used to differentiate patients with AD from individuals with normal cognitive abilities (Olazarán et al., 2015). By measuring the levels of primary and secondary bile acid metabolites in the serum, it is possible to evaluate the presence of amyloid protein, Tau, and neurodegeneration in AD (Nho et al., 2019). Similar metabolomics could enable the earlier detection and intervention if it can be applied to identify preclinical cognitive impairment before the manifestation of obvious clinical symptoms (Wang et al., 2020). Moreover, as we mentioned earlier, the identification of bile acids and their receptors within the brain leads to the inference that they potentially influence neurological functions directly (Mulak, 2021). Numerous studies have presented compelling evidence regarding the significant correlation between altered bile acid profiles and cognitive decline (MahmoudianDehkordi et al., 2019). Untargeted fecal metabolomics analysis has provided evidence that the therapeutic management of AD could potentially involve the regulation of bile acid biosynthesis metabolism (Wang A. et al., 2022). The aforementioned evidence indicates a close connection between bile acids and the onset and progression of AD. Drawing from the current body of evidence, it can be inferred that the regulation of bile acids to elicit neuroprotective effects might offer novel avenues for both preventing and treating AD.

## 3 Characteristics of TUDCA

Tauroursodeoxycholic acid (TUDCA) or ursodeoxycholic acid (UDCA) has displayed therapeutic potential in treating neurodegenerative diseases (Vang et al., 2014; Khalaf et al., 2022). TUDCA was first identified in bear bile in 1902, which had been utilized in traditional Chinese medicine for centuries due to its believed abilities to alleviate heat, detoxify, calm wind, stop spasms, and enhance liver and ocular health (Wang, 2014). TUDCA is categorized as a hydrophilic bile acid and falls under the classification of endogenous conjugated bile acids. It is formed through the bonding of the amino group of taurine with the carboxyl group of UDCA. Initially, the United States Food and Drug Administration (FDA) approved UDCA for the treatment of specific cholestatic diseases (Vang et al., 2014; Kusaczuk, 2019). TUDCA, in addition to its considerable therapeutic benefits and capacity to cross the blood–brain barrier, offers favorable tolerance and safety profiles (Elia et al., 2016), leading to its widespread use in clinical settings. In addition to manual extraction from bear bile, chemical synthesis serves as the primary source of TUDCA (Dayal et al., 1996). Previous research has elucidated that TUDCA has the ability to protect nerves by regulating cell apoptosis (Amaral et al., 2009). Various studies, ranging from cell and animal models to clinical trials, provide substantial evidence supporting the potential effectiveness of TUDCA in treating several neurodegenerative diseases (Ackerman and Gerhard, 2016). Its application in Parkinson’s disease (PD), amyotrophic lateral sclerosis (ALS), diabetes retinopathy and other disease models has shown promising results in effectively inhibiting pathological changes (Gaspar et al., 2013; Daruich et al., 2019; Thams et al., 2019; Cuevas et al., 2022).

## 4 The therapeutic effect and mechanism of TUDCA on Alzheimer’s disease

**Figure 1:**
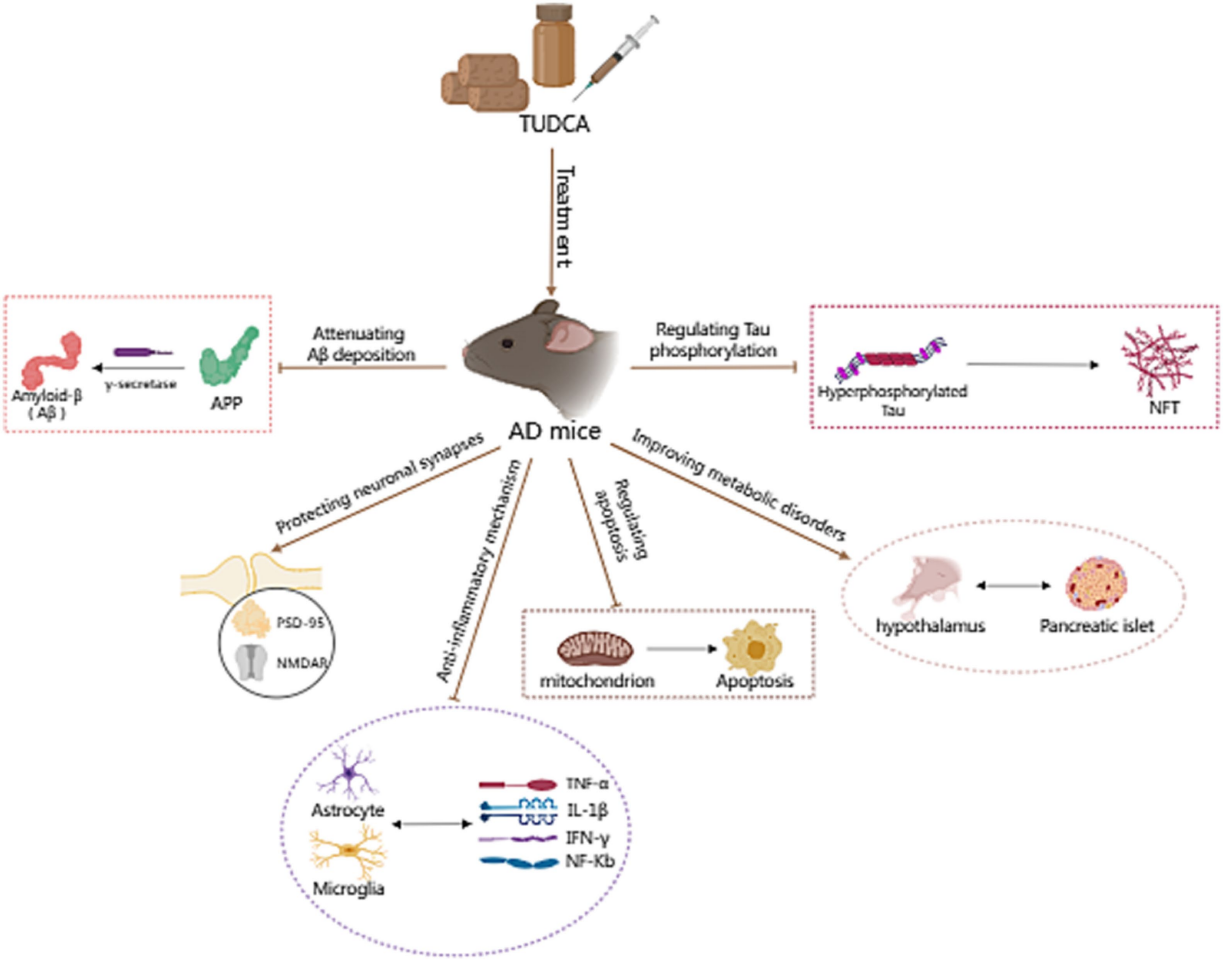
The potential mechanism of TUDCA in treating AD. The potential mechanisms of TUDCA in treating AD may be related to reducing Amyloid-β protein deposition, regulating apoptotic pathways, preventing tau hyperphosphorylation and aggregation, protecting neuronal synapses, exerting anti-inflammatory effects, and improving metabolic disorders. The therapeutic effects of TUDCA are represented by brown arrows, while the brown arrows with flat heads indicate the inhibitory effects on specific pathways.

Increasing evidence suggests that TUDCA may effectively inhibit the development and advancement of Alzheimer’s disease (AD; Zangerolamo et al., 2021b), and has the potential to both prevent and treat cognitive impairment. Lo et al. (2013) conducted a randomized controlled trial, wherein AD model mice, who had not yet developed the disease, and wild type mice were assigned randomly to either a diet containing 0.4% TUDCA (as a sodium salt) or regular diet, for a duration of 6 months. Following the intervention, the mice in each group were subjected to assessments through the Morris water maze, Social recognition, and Passive avoidance tests to evaluate their respective performances. The findings revealed a significant improvement in spatial learning abilities and contextual ear learning, as well as enhanced recognition memory, following TUDCA treatment. These results confirm that TUDCA treatment in the AD mouse model can effectively hinder the progression of cognitive impairment, while not adversely affecting the cognitive function of normal wild-type mice. Another study has demonstrated that the insufficient discrimination between conditioned and novel contexts in AD mice can be reversed through treatment with TUDCA, and this treatment also helps prevent memory deficits (Nunes et al., 2012). In the study conducted by Dionísio et al. (2015), TUDCA was injected to mice at a dosage of 500 mg/kg of body weight after the onset of AD, every 3 days, for a period of 3 months. The Morris water maze test results showed that control AD mice did not exhibit a preference for the target quadrant, whereas the mice in the TUDCA-treated group showed a trend toward improved memory. These findings suggests that TUDCA may hold promise as a viable therapeutic strategy for both preventing and treating AD, even after the disease has already manifested. Previous studies have established that TUDCA can effectively prevent learning and memory impairments in AD mice. However, the specific mechanisms responsible for this effect remain largely unclear, which may be related to reducing Amyloid-β protein deposition, regulating apoptotic pathways, preventing tau hyperphosphorylation and aggregation, protecting neuronal synapses, exerting anti-inflammatory effects, and improving metabolic disorders (Figure 1).

### 4.1 Reducing amyloid-β deposition in brain

Amyloid-β (Aβ) imbalance in generation and clearance is the initiating factor for the occurrence of dementia (Baker-Nigh et al., 2015). Aβ is deposited in specific areas of the brain, leading to a series of pathological changes such as neuronal degeneration, inflammation, and glial proliferation, and affecting cognitive and other brain functions (Selkoe, 2001). It is widely accepted that the deposition of Aβ in the brain is a pathological characteristic of Alzheimer’s disease (Bero et al., 2011), and the accumulation of Aβ peptides plays a critical role in the pathogenesis of AD (Nunes et al., 2012). Aβ peptides derive from the sequential proteolysis of the amyloid precursor protein (APP; Haass et al., 2012), which necessitates the participation of γ-secretase (Edbauer et al., 2003). Furthermore, mutations in the genes responsible for coding APP and presenilin 1 (PS1), the catalytic core of γ-secretase, are, respectively, associated with increased amyloidogenic processing of APP and preferential production of longer Aβ species with higher amyloidogenic propensity (Selkoe, 2002). The APP/PS1 double-transgenic mouse model is a well-established model for Alzheimer’s disease (Radde et al., 2006). Studies have demonstrated that this model develops AD neuropathology at the age of 2 months, while behavioral impairments start to occur from 8 months of age and continue thereafter. Nunes et al. (2012) conducted a study in which two-month-old APP/PS1 mice were treated with TUDCA for a period of 6 months. The mice were given a diet consisting of regular laboratory chow, with the addition of 0.4% (wt/wt) TUDCA (sodium salt). Immunohistochemistry analysis confirmed a significant decrease of approximately 65% in Aβ plaque number in the hippocampus, and a 40% decrease in the frontal cortex of the APP/PS1 mice that were treated with TUDCA, in comparison to the transgenic mice that did not receive treatment. Additionally, the APP/PS1 mice that received TUDCA showed a decrease in CTF-γ production according to the results obtained from immunoblot analysis, when compared to the transgenic mice in the control group. This suggests that the activity of γ-secretase is modulated by TUDCA, which in turn affects the processing of APP. Another randomized controlled study also yielded similar results, where 2-month-old APP/PS1 mice were administered TUDCA treatment for 6 months. The study revealed a decrease in the number of amyloid plaques in the prefrontal cortex and hippocampus in comparison to regular APP/PS1 mice (Lo et al., 2013). This reduction was consistent with an observed improvement in cognitive function. In addition, after receiving an injection of 500 mg/kg body weight of TUDCA or vehicle, a significant reduction in the number of amyloid plaque in the hippocampus was observed, and the amyloid burden in both the hippocampus and frontal cortex was decreased in 7-month-old APP/PS1 mice (Dionísio et al., 2015). The aforementioned results indicate that TUDCA has potential in the prevention and alleviation of AD, particularly in the context of Amyloid-β pathology. In order to gain a clearer understanding of the effects of TUDCA on Amyloid-β pathology, Aβ peptides have been co-incubated with TUDCA *in vitro* (Viana et al., 2009). It is noteworthy that this co-incubation resulted in no significant changes in the aggregation or secondary structures of the Aβ peptides. These findings provide support for the idea that TUDCA influences APP processing and Aβ production, rather than affecting fibrillogenesis.

**Figure 2:**
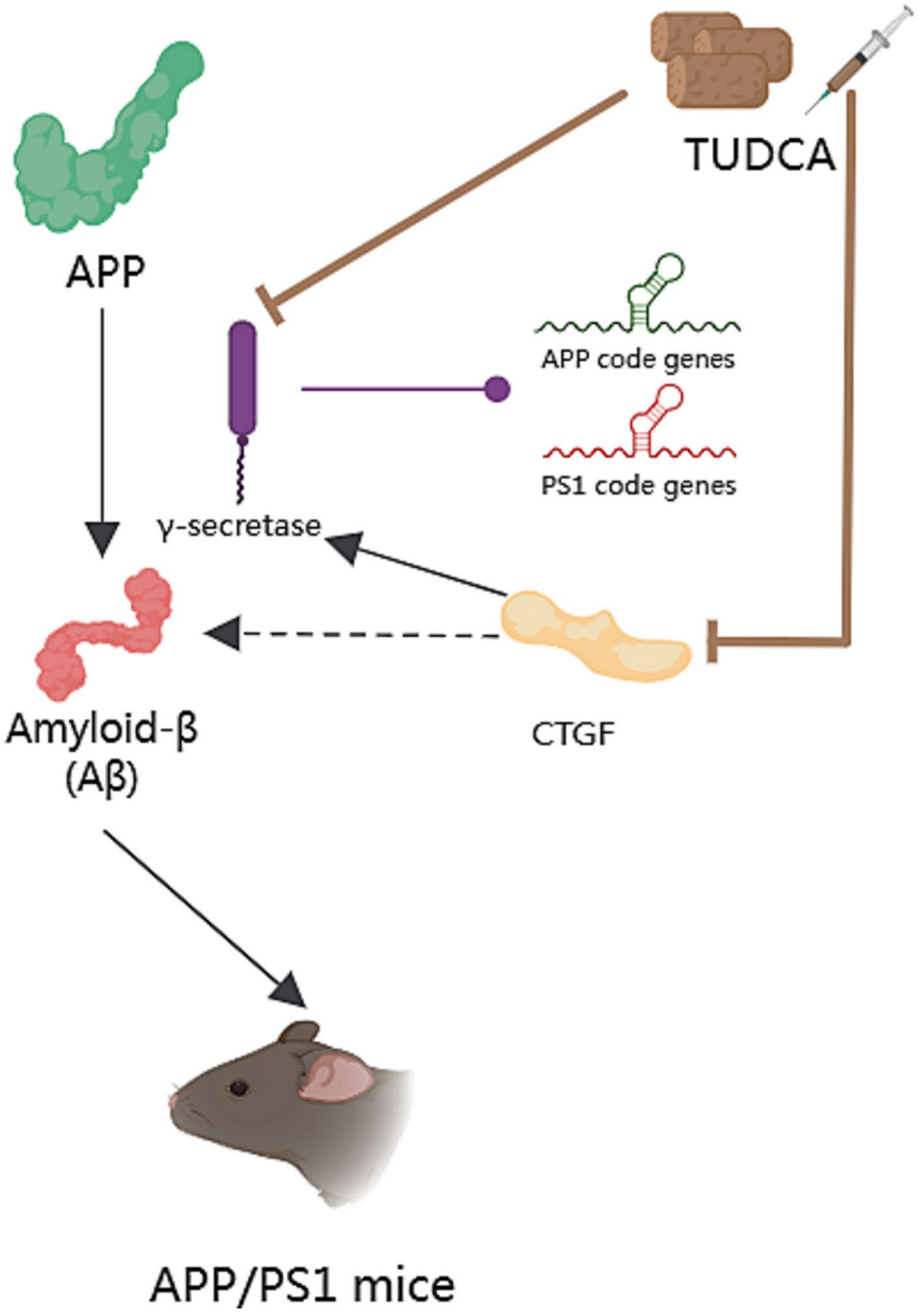
The effect of TUDCA in reducing Amyloid-β deposition. Amyloid-β (Aβ) peptides derive from the sequential proteolysis of the amyloid precursor protein (APP) and induce AD, as shown by the black arrows in the figure. This process necessitates the participation of γ-secretase, which could catalyze mutations in the genes responsible for coding APP and presenilin 1 (PS1), as indicated by the arrow with a purple dot. The activity of γ-secretase can be modulated by TUDCA, which subsequently affects the processing of APP and reduces Aβ deposition. TUDCA could also lead to a downregulation of connective tissue growth factor (CTGF), a known enhancer of γ-secretase activity and Aβ neuropathology. The inhibitory effects of TUDCA on γ-secretase and CTGF are represented by two brown arrows with flat heads. Additionally, the black arrow signifies the activation effect of CTGF on γ-secretase, while the black dashed arrow indicates the correlation between CTGF and Aβ.

Studies have shown that the expression of connective tissue growth factor (CTGF) correlates with the progression of amyloid neuritic plaque neuropathology (Zhao et al., 2005), and CTGF expression is increased near Aβ plaques in post-mortem AD brains (Ueberham et al., 2003). CTGF is a cysteine-rich protein, closely related to the occurrence of fibrosis in tissues and organs like the liver. Additionally, CTGF is known to enhance γ-secretase activity and Aβ neuropathology (Lo et al., 2013). In the study of Nunes et al. (2012), it was observed that APP/PS1 mice displayed heightened CTGF levels in the brain compared with wild-type mice. However, treatment with TUDCA led to a downregulation of CTGF in the hippocampus and frontal cortex of APP/PS1 mice. A reduction in APP-CTF-γ levels coincided with the decrease in CTGF expression. The study findings indicate that TUDCA may reduce the expression of CTGF and regulate γ-secretase activity to inhibit APP processing, leading to a decrease in Aβ deposition and interference with AD pathology (Figure 2).

### 4.2 Modulating apoptosis in AD

Apoptosis is a general mechanism of cellular demise in Alzheimer’s disease. Early investigations in the brains of AD patients displayed a significant increase in apoptosis, approximately 50 times more than age-matched controls (Colurso et al., 2003). A vital role in the induction of apoptosis caused by Aβ is played by tumor suppressor protein p53, which is well-known for its capability to increase the levels of apoptotic proteins including Bax. Existing evidence suggests that exposure to Aβ makes PC12 cells show an increase in E2F-1 expression, and increases levels of p53 and Bax, culminating in nuclear fragmentation (Ramalho et al., 2004). Similar findings were observed in neuroblastoma cells upon inducing endogenous expression of Aβ (Ramalho et al., 2006). Furthermore, both transgenic mice and the human AD brain exhibited an accumulation of p53 in degenerating neurons (Ramalho et al., 2008). These findings highlight the significant involvement of p53-dependent neuronal apoptosis in the pathological development of AD. Importantly, TUDCA has been proven to increase the threshold for apoptosis in various cell types. When PC12 neuronal cells were incubated with TUDCA, it effectively inhibited the induction of E2F-1, stabilization of p53, and expression of Bax that were induced by Aβ. Moreover, TUDCA provided protection to PC12 cells against apoptosis that was caused by the overexpression of E2F-1 and p53. An *in vitro* model of familial Alzheimer’s Disease (AD) has provided further confirmation that TUDCA has the ability to modulate p53-mediated apoptosis in AD (Ramalho et al., 2006). In neuroblastoma cells, TUDCA could modulate the activity of p53 and the alterations in the Bcl-2 family. TUDCA’s regulation of p53 appears to involve the activation of a survival pathway mediated by the PI3K enzyme in neurons and the activation of protein kinase AKT (Ramalho et al., 2006). Additionally, TUDCA has been found to prevent caspase-2 activation, which could trigger apoptosis through the mitochondrial pathway (Ramalho et al., 2008).

**Figure 3:**
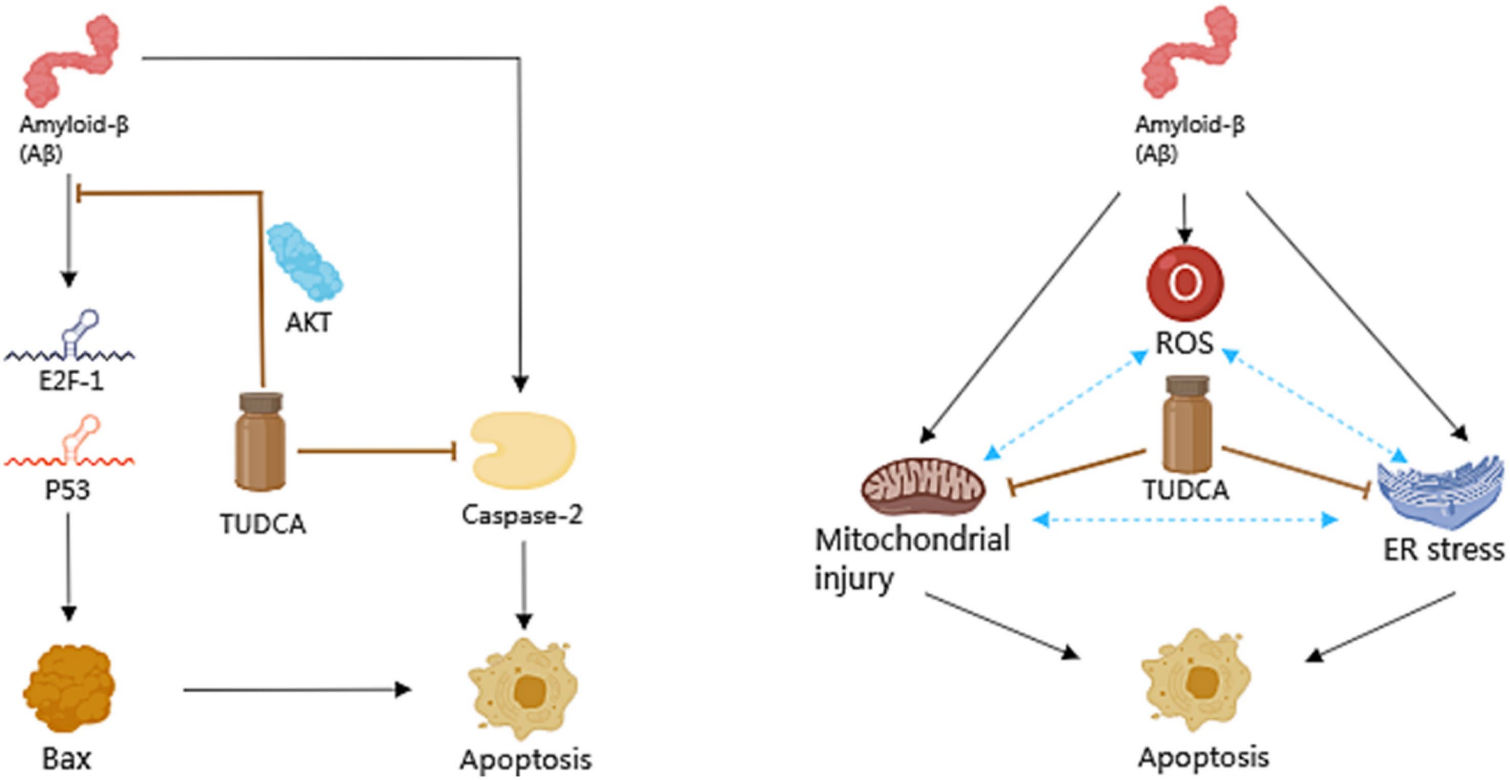
The role of TUDCA in modulating apoptosis in AD. TUDCA could effectively inhibit the induction of E2F-1, stabilization of p53, and expression of Bax, all of which are typically induced by Aβ. This effect appears to occur through the activation of protein kinase AKT. Additionally, TUDCA has the ability to inhibit apoptosis by preventing caspase-2 activation. Furthermore, Aβ has been found to cause dysfunction of mitochondria, endoplasmic reticulum (ER) stress and the overproduction of reactive oxygen species (ROS), all of which play significant roles in the process of apoptosis in AD. These factors could create a detrimental cycle that exacerbates AD pathology, as shown by three blue bidirectional dashed arrows. However, TUDCA can help ameliorate mitochondrial dysfunction and reduce ER stress, thus providing further modulation of apoptosis in AD. The black arrows represent the pathways involved in apoptosis, while the inhibitory effects of TUDCA on these pathways are indicated by brown arrows with flat heads.

There is a growing body of evidence indicating that both the dysfunction of mitochondria and endoplasmic reticulum (ER) stress play significant roles in the process of apoptosis in Alzheimer’s disease. Mitochondria are intimately associated with the process of cellular apoptosis, and proper functioning of mitochondria is essential for maintaining cellular health (Kasahara and Scorrano, 2014). ER stress is a protective stress response, which could activate the caspase-12 mediated apoptosis pathway. Moreover, it has been found to play a role in causing the death of neurons. It is worth noting that Aβ has the capability to directly or indirectly harm mitochondria (Ramalho et al., 2008), and the accumulation of Aβ and Tau has been proposed to cause ER stress (Ochiai et al., 2021). Additionally, by blocking both the respiratory complex I and pyruvate dehydrogenase, Aβ stimulates the overproduction of reactive oxygen species (ROS; Ramalho et al., 2008), which contribute to oxidative damage. Moreover, ER stress has been shown to have an additional effect on the production of ROS. The aforementioned processes ultimately lead to mitochondrial dysfunction, impaired energy metabolism, and activation of apoptotic pathways, thereby exacerbating AD pathology, and forming a vicious cycle. After incubating with TUDCA, the inhibition of Aβ-induced mitochondrial membrane permeabilization and the subsequent release of cytochrome c were observed in neuronal mitochondria that were isolated. Furthermore, TUDCA could prevent changes in the redox status of the mitochondrial membrane, polarity of lipids, and order of proteins driven by Aβ (Ramalho et al., 2008). At the same time, research shows that TUDCA can alleviate central ER stress in APP/PS1 mice (Ochiai et al., 2021). To sum up, TUDCA has the potential to inhibit apoptosis in AD by regulating p53 expression, ameliorating mitochondrial dysfunction, and reducing ER stress (Figure 3).

### 4.3 Preventing hyperphosphorylation and aggregation of tau

Neurofibrillary tangles (NFTs), consist of hyperphosphorylated and aggregated microtubule-associated protein Tau, is another pathological hallmark of Alzheimer’s disease (Zangerolamo et al., 2021a). Tau is a hydrophilic protein, with its longest isoform (2N4R) containing 80 Ser or Thr residues, 56 negative (Asp or Glu) residues, 58 positive (Lys or Arg) residues and 8 aromatic (5 Tyr and 3 Phe, but no Trp) residues (Wang and Mandelkow, 2016). There are as many as 85 potential phosphorylation sites (80 Ser or Thr, and 5 Tyr) in the 2N4R isoform (Hanger et al., 2009). The latest research indicates that three key residues (Thr50, Thr69, and Thr181) described as master sites and two kinases (GSK3β and p38α) are closely related to the regulation of Tau protein phosphorylation levels (Stefanoska et al., 2022). Phosphorylation plays a crucial part in regulating the physiological functions of Tau. It has been validated that phosphorylation sites could alter the stability of the Tau-microtubule complex (Brotzakis et al., 2021). Previous studies have confirmed that phosphorylation of Ser262 and Ser396 contributes to reduced binding of tau for microtubules (Biernat et al., 1993; Bramblett et al., 1993). Similarly, phosphorylation of Ser214 and Thr231 in the flanking region of Tau can trigger the detachment of Tau from microtubules. In addition, Tyr phosphorylation might contribute to Tau aggregation (Wang and Mandelkow, 2016). In summary, phosphorylation induces Tau to lose its ability to bind to microtubules and promotes the aggregation of Tau. It is important to note that Tau is also influenced by other post-translational modifications (PTMs; Martin et al., 2011). PTMs have been implicated in influencing Tau aggregation and the development of AD, as indicated by a significant post-mortem proteomic analysis of tau in AD patients’ brains vs. control brains (Wesseling et al., 2020). For instance, glycation of tau may reduce the binding of tau to microtubules. Furthermore, nitration of Tyr residues alters the conformation of tau, resulting in a reduced binding to microtubules. Depending on the specific sites of nitration, it can either promote or inhibit tau aggregation (Reyes et al., 2012). Additionally, acetylation, methylation and ubiquitylation of Lys residues can affect the phosphorylation, aggregation and degradation processes of Tau (Petrucelli, 2004; Funk et al., 2014; Min et al., 2015). Despite the existence of diverse post-translational modifications of Tau, current studies on AD primarily focus on the phosphorylation of Tau protein.

Previous research has indicated that APP/PS1 mice present hyperphosphorylated Tau-positive neuritic structures located in the proximity of amyloid plaques at 8 months of age (Radde et al., 2006). In another study, p-tau levels in the hippocampus and frontal cortex of APP/PS1 mice were found to be increased approximately 2-fold relative to those in control wild-type mice (Dionísio et al., 2015). In recent years, clinical studies have shown that abnormal phosphorylation of the Tau protein occurs prior to the accumulation of amyloid-β in the brain (Ye et al., 2024). All of these findings collectively support the notion that abnormally hyperphosphorylated Tau is crucial in the progression of AD. At the same time, animal models such as the APP/PS1/Tau triple transgenic (3 × Tg-AD) mice which could further induce abnormal phosphorylation of Tau (Li et al., 2019; Wang et al., 2021), and the hTau mice with murine Tau knockout background (Hu et al., 2016; Li et al., 2022), have also been utilized to explore the therapeutic potential of inhibiting pathological alterations of Tau in AD. The excessive phosphorylation of Tau could lead to the accumulation of Tau protein, causing ER stress, synaptic dysfunction, and neurodegeneration (Ochiai et al., 2021), which can exacerbate the progression of AD. Thus, reducing the levels of Tau protein hyperphosphorylation can effectively alleviate AD (Dai et al., 2017). It is worth noting that Tau pathology is linked to the activation of multiple apoptotic signals in AD. Research has shown that the activation of caspase-3 by Aβ triggers the cleavage and aggregation of Tau (Rissman et al., 2004). Furthermore, the overexpression of p53 is linked to the indirect triggering of abnormal phosphorylation of Tau (Hooper et al., 2007). TUDCA has been found to possess the potential to enhance neuronal survival by decreasing caspase-3 activation and the subsequent cleavage of tau protein (Ramalho et al., 2008). Meanwhile, TUDCA has the ability to promote the signaling pathway known as PI3K/Akt in AD (Solá et al., 2003), leading to the inhibition of p53 expression and subsequent regulation of Tau phosphorylation.

Importantly, Glycogen synthase kinase 3β (GSK3β) is also a substrate of Akt (Beaulieu et al., 2009), Akt has the ability to phosphorylate GSK3β and inhibit its activity. And it is recognized that GSK3β phosphorylates Tau and contributes to the formation of NFTs, as described above. Interestingly, Dionísio et al. (2015) demonstrated a decrease in Akt activity in the frontal cortex of APP/PS1 mice compared to littermates from the control group. However, there were no notable changes detected in the hippocampus. On the other hand, GSK3β was found to be excessively activated in both brain regions, suggesting that the dysregulation of GSK3β in the hippocampus is likely independent of the upstream Akt pathway. The treatment of TUDCA could activate Akt in both the frontal cortex and the hippocampus. Accordingly, the decrease in p-GSK3b levels observed in APP/PS1 mice in both brain regions was reversed with TUDCA treatment. At the same time, transgenic animals that were treated with TUDCA showed a significant decrease in p-tau levels in both brain regions. The decrease amounted to approximately 40% compared to the control group of APP/PS1 mice. This study provides evidence that TUDCA’s inhibitory influence on GSK3β activity has a positive impact on Tau hyperphosphorylation, and this effect is associated with increased Akt activity.

**Figure 4:**
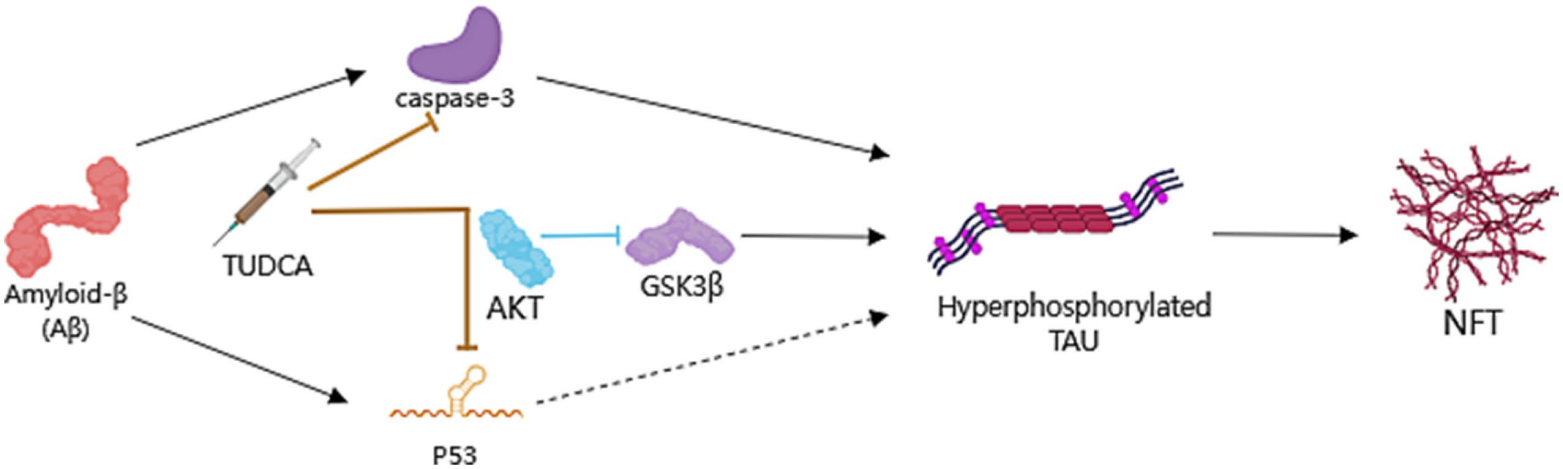
TUDCA’s impact on Preventing hyperphosphorylation and aggregation of Tau. Neurofibrillary tangles (NFTs), consist of hyperphosphorylated and aggregated microtubule-associated protein Tau, is another pathological hallmark of AD. TUDCA could decrease Aβ-induced activation of caspase-3 and the subsequent cleavage of Tau protein, while promoting the PI3K/Akt signaling pathway in AD to inhibit p53 expression and the activity of Glycogen synthase kinase 3β (GSK3β), thereby regulating Tau phosphorylation. The processes of induction and formation of hyperphosphorylated Tau and NFTs are represented by black arrows. The inhibitory effects of TUDCA on caspase-3 and p53 are indicated by two brown arrows with flat heads, while the inhibitory effects of AKT on GSK3β are indicated by a blue arrow with a flat head.

In summary, TUDCA has been shown to potentially reduce activation of caspase-3 while simultaneously activating the PI3K/Akt signaling pathway. This activation ultimately results in the inhibition of the expression of p53 and GSK3β activity. Taken together, these effects appear to assist in the regulation of Tau phosphorylation and aggregation (Figure 4). However, existing research has mainly focused on the use of TUDCA in APP/PS1 mice, lacking other models that is relevant to tau. Additionally, the exploration of the relationship between TUDCA and Tau has been limited in its depth, leading to certain limitations in the conclusions drawn. Therefore, further research is necessary to investigate the impact of TUDCA on other Tau PTMs and kinases involved in Tau hyperphosphorylation. This will help to provide a clearer understanding of the effects of TUDCA.

### 4.4 Protecting neuronal synapses

Synapses are the functional connections between neurons, responsible for information transmission, and closely related to the formation of memory (Underwood et al., 2023). It was reported that the density of synapses in the cortical and hippocampal regions is decreased during the early stages of Alzheimer’s disease (Gouras et al., 2010). Furthermore, both synthetic Aβ oligomers and natural soluble oligomeric Aβ have been found to have detrimental effects on synapses (Ramalho et al., 2013). The AD mouse model also showed changes in synaptic transmission and plasticity before the onset of neuronal demise and plaque formation. These findings provide compelling evidence that synapses play a crucial role as the early pathological site in AD. Moreover, the influence of Aβ oligomers on synaptic plasticity is an important contributor to the development of AD, as it can hinder long-term potentiation (LTP), a type of synaptic plasticity that is vital for the processes of memory and learning. Additionally, these oligomers can also contribute to the induction of long-term depression (LTD) in hippocampal synapses (Shankar et al., 2008). Disruption to these mechanisms of synaptic plasticity results in loss of memory and a decline in cognitive function in AD. Selkoe et al.’s research further supports this notion by demonstrating that Aβ oligomers possess the capability to negatively impact synaptic plasticity, structure and the memory of a complex learned behavior (Selkoe, 2008). These discoveries emphasize the significance of synaptic plasticity and physiology in the progression of AD. Synaptic loss proves to be the most prominent pathological factor associated with cognitive decline in AD.

The postsynaptic density terminals play a vital role in the creation and retention of memories (Sjöström et al., 2008). These sites within the neurons are integral for the encoding, consolidation, and retrieval of information that contributes to long-term memory formation. Consequently, postsynaptic structural proteins have been proposed as the most pertinent indicators for assessing the progression of AD. The postsynaptic density-95 protein (PSD-95) serves as a scaffold protein at the postsynaptic site and has been described to be downregulated in transgenic mouse models of AD (Shao et al., 2011). Ramalho et al. (2013) discovered that levels of PSD-95 in isolated rat neurons decreased by approximately 50% after being incubated with the active fragment Aβ25-35 for 12 h. However, when incubated with TUDCA, PSD-95 levels were restored to almost the same as control levels. Additionally, TUDCA was effective in preventing neuronal death caused by incubation with the active fragment Aβ25-35. Furthermore, in the hippocampus of mice carrying mutations in the APP and PS1 genes, PSD-95 reactivity was reduced, but the transgenic mice that were treated with TUDCA showed an inhibition of approximately 70% in the decrease, in contrast to untreated APP/PS1 controls. Moreover, the modulation of synaptic density by TUDCA revealed that TUDCA has the potential to inhibit Aβ-induced synaptic changes both in laboratory settings and in living organisms, and protect neurons. Additional research has further confirmed the neuroprotective effect of TUDCA on neurons (Nunes et al., 2012) and synapses (Dionísio et al., 2015).

Interestingly, TUDCA may also improve synaptic plasticity. Excitatory synapses’ structural and functional integrity is determined by PSD-95, which clusters with postsynaptic excitatory receptors, specifically the N-methyl-D-aspartate receptor (NMDAR; Chen et al., 2008). Synaptic NMDARs are required for LTP, while extrasynaptic NMDARs have the ability to initiate *de novo* LTD. Aβ oligomers could reduce synaptic NMDAR activation and enhance extrasynaptic NMDARs. This leads to the promotion of LTD and inhibition of LTP signaling mediated by NMDARs (Shankar et al., 2007; Li et al., 2011). TUDCA has been demonstrated to prevent the decrease in PSD-95 expression both *in vitro* and in APP/PS1 transgenic mice. This suggests that TUDCA may regulate the effects of Aβ on the NMDA Type Glutamate Receptor-Dependent Signaling Pathway. In addition, TUDCA’s ability to prevent changes in spontaneous synaptic activity also indicates that it may improve presynaptic glutamate release impaired by Aβ (Ramalho et al., 2013).

**Figure 5:**
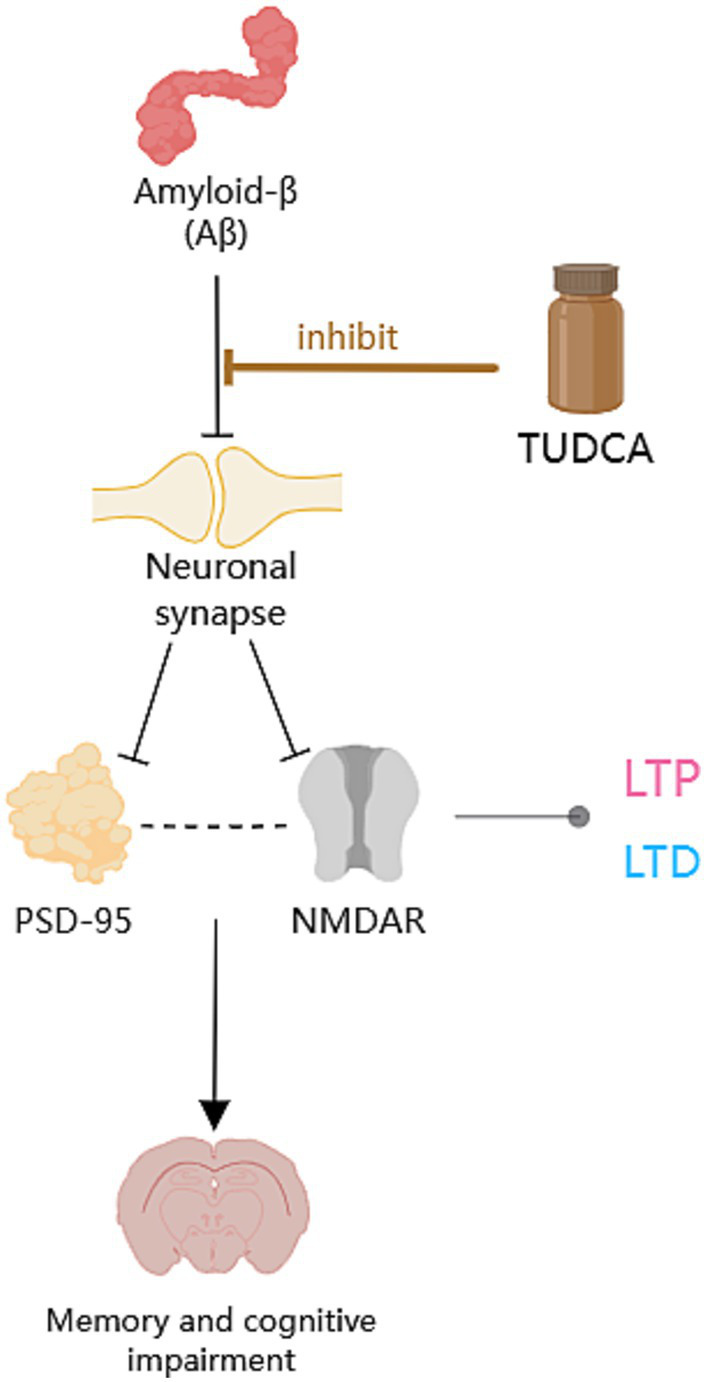
The protective role of TUDCA on neuronal synapses. Aβ has detrimental effects on synapses, as highlighted by the occurrence of multiple black arrows with flat heads. Aβ could downregulate the postsynaptic density-95 protein (PSD-95) and affect postsynaptic excitatory receptors that clusters with PSD-95, specifically the N-methyl-D-aspartate receptor (NMDAR). The correlation between PSD-95 and NMDAR is depicted by a dashed arrow, shown in black. Moreover, Aβ could hinder long-term potentiation (LTP) and contribute to the induction of long-term depression (LTD), both of which are types of synaptic plasticity related to NMDAR, denoted by an arrow accompanied by a gray dot. However, TUDCA may inhibit Aβ-induced synaptic structural changes and neuronal degeneration, as indicated by a brown arrow with a flat head, while also affecting synaptic plasticity to promote the recovery of memory and cognitive impairment.

The aforementioned research suggests that TUDCA may inhibit Aβ-induced synaptic structural changes and neuronal degeneration, as well as affect synaptic plasticity through its impact on the NMDA Type Glutamate Receptor-Dependent Signaling Pathway, to promote the recovery of AD (Figure 5).

### 4.5 Anti-inflammatory mechanisms

**Figure 6:**
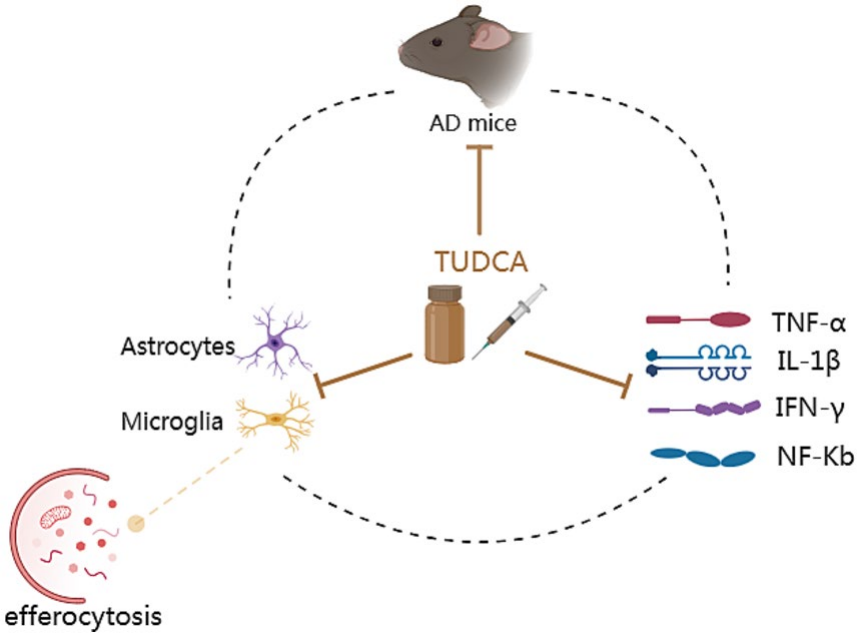
The anti-inflammatory mechanisms of TUDCA. Inflammation exacerbation is one of the characteristics of AD. AD mouse models exhibit elevated levels of inflammatory cytokines such as TNF-α, IL-1β, IFN-γ and NF-Κb. These increased inflammatory signals contribute to the worsening of AD pathology. AD can also result in the activation of astrocytes and microglia, as well as the damage of efferocytosis (the process responsible for eliminating programmed cell death and potentially exerting anti-inflammatory effects), contributing to the progression of an inflammatory process. Furthermore, microglia activation could lead to the accumulation of Aβ. These pathological changes form a vicious cycle, as depicted by a dashed black circle. Significantly, TUDCA possesses the capability to simultaneously exert effects on all of them, as indicated by multiple brown arrows with flat heads. In summary, TUDCA has the ability to regulate the activation of glial cells, efferocytosis and inflammatory response, thereby preventing the further development of AD pathology.

Inflammation exacerbation is one of the characteristics of AD and has a fundamental impact on the progression of the disease (Kinney et al., 2018), contributing to neuronal degeneration and loss. Elevated inflammatory signals can promote the expression of APP and increase the activity of γ-secretase, leading to the release of a large amount of Aβ peptide (Decourt et al., 2017), which worsens AD pathology. Previous research has demonstrated that AD mouse models exhibit elevated levels of inflammatory cytokines such as TNF-α, IL-1β, and IFN-γ. However, there was a notable decrease in the levels of inflammatory cytokines expressed after a 10-day treatment of TUDCA (300 mg/kg) injection (Zangerolamo et al., 2021a). The escalation of the inflammatory response following AD is intricately connected to the activation of glial cells. Both AD patients and mice models exhibited activation of astrocytes and microglia in the area where Aβ plaques were present in their brains, contributing to the progression of an inflammatory process in the injured region of the brain (Nunes et al., 2012). Importantly, TUDCA treatment has been found to effectively suppress astrocyte and microglial activation in mice with the APP/PS1 genetic mutation. Furthermore, it has shown potential in attenuating the production of inflammatory markers, including NF-Κb and TNF-α (Nunes et al., 2012; Ramalho et al., 2013; Dionísio et al., 2015). Furthermore, microglia activation could impair phagocytosis leading to the accumulation of Aβ (Krabbe et al., 2013), and anti-TNF-a therapeutic strategies have been found to decrease amyloid deposition and tau hyperphosphorylation (Shi et al., 2011). These studies further establish a connection between TUDCA and Aβ/tau. The efferocytosis of microglia presents a potentially effective therapeutic target. Unlike phagocytosis, efferocytosis exerts anti-inflammatory effects and aids in tissue repair. Cash et al. (2012) conducted a study that demonstrated the ability of TUDCA to reverse the efferocytosis damage induced by APOE4. In conclusion, TUDCA possesses the ability to regulate the activation of glial cells and inflammatory response caused by AD pathology. Moreover, it may exhibit an anti-inflammatory effect by restoring efferocytosis (Figure 6).

### 4.6 Improving metabolic disorders

Abnormal energy metabolism and hypothalamic dysfunction have been found to be associated with Alzheimer’s disease (Clarke et al., 2015). On the one hand, hypothalamic dysfunction can affect the progress of AD and contribute to its pathogenesis (Vercruysse et al., 2018); On the other hand, AD-induced mitochondrial dysfunction and ER stress can intensify the burden on organs like the hypothalamus, thereby further impairing metabolism (Ochiai et al., 2021). As mentioned earlier, metabolic disorders link AD to diabetes, obesity and other diseases. Surveys have implied that 80% of individuals with AD exhibit impaired glucose tolerance or have diabetes (Janson et al., 2004). Furthermore, individuals with type 2 diabetes are at a higher risk of being diagnosed with dementia, with the likelihood being 1.5 to 2 times greater (Biessels et al., 2014). Studies have shown that insulin resistance in type 2 diabetes mellitus (T2DM) contributes to pathological changes including Aβ plaque deposition (Beeler et al., 2009). Additionally, impaired glucose tolerance and insulin resistance have been observed in mice models of AD (Macklin et al., 2017). Intracerebroventricular (ICV) injection of Streptozotocin (STZ), a chemical used to induce both type 1 and type 2 diabetes mellitus in rodents, can be used to create a mouse model of AD. This model exhibits reduced brain weight and increased levels of amyloid oligomers. Moreover, the Streptozotocin-induced AD mouse model also displays increased body weight, along with impaired glucose tolerance and insulin resistance (Zangerolamo et al., 2021c). After receiving an intraperitoneal dose of 300 mg/kg of TUDCA for a duration of 10 days, the body weight and adiposity of STZ-induced AD mice were observed to decrease. Furthermore, the results showed a significant increase in glucose tolerance and insulin sensitivity as well. TUDCA treatment could also increase islet mass, β-cell area and insulin secretion in response to glucose stimulation. Importantly, TUDCA-treated STZ mice also exhibited reduced neuroinflammation and decreased amyloid oligomers’ protein content. In addition, BDNF mRNA was observed to be present in higher levels in the hippocampus, along with improved results on memory tests. Moreover, the treatment with TUDCA reversed the decrease in insulin receptor (IR) β-subunit protein expression caused by AD pathogenesis in the hippocampus. This suggests that TUDCA potentiates the neuroprotective effect of insulin in this area (Zangerolamo et al., 2021c).

Other studies have also explored the correlation between TUDCA and metabolic disorders, especially diabetes. For example, type II diabetes is related to the aggregation of islet amyloid polypeptide (IAPP), which leads to a decline in pancreatic β-cell function and mass (Roham et al., 2022). What’s more, IAPP and amyloid-β share many biophysical and physiological properties. They also possess comparable cytotoxic mechanisms, which could lead to the development of AD and a decline in cognitive function (Zhang and Song, 2017; Ferreira et al., 2021). Therefore, targeting IAPP could prove to be beneficial not only for managing type II diabetes but also for potentially treating or even preventing AD (Bortoletto and Parchem, 2023). Significantly, the chemical chaperone TUDCA has been shown to alleviate IAPP-induced ER stress, increase insulin secretion in IAPP-expressing cells, and ameliorate β-cell dysfunction (Cadavez et al., 2014). In addition, TUDCA could effectively stimulate the release of glucagon-like peptide-1(GLP-1) by activating the bile acid receptor TGR5 located on L-cells, which are a particular group of enteroendocrine cells (EECs; Brighton et al., 2015). GLP-1 has demonstrated its efficacy in treating diabetes and obesity by utilizing multiple mechanisms, including stimulating insulin secretion, inhibiting glucagon secretion, protecting β cells and decreasing food intake. Additionally, it has the potential to impact neurological and cognitive functions (Yildirim Simsir et al., 2018). By manipulating GLP-1 activity, the aggregation of amyloid-β in AD can be regulated, and GLP-1 receptor agonists could alleviate hippocampal neurodegeneration (Batista et al., 2019). To sum up, based on the evidence that TUDCA has become an emerging anti-diabetic drug (Ajmal et al., 2023), investigating how TUDCA can impact AD by improving metabolism could potentially extend the therapeutic benefits of TUDCA beyond just AD.

**Figure 7:**
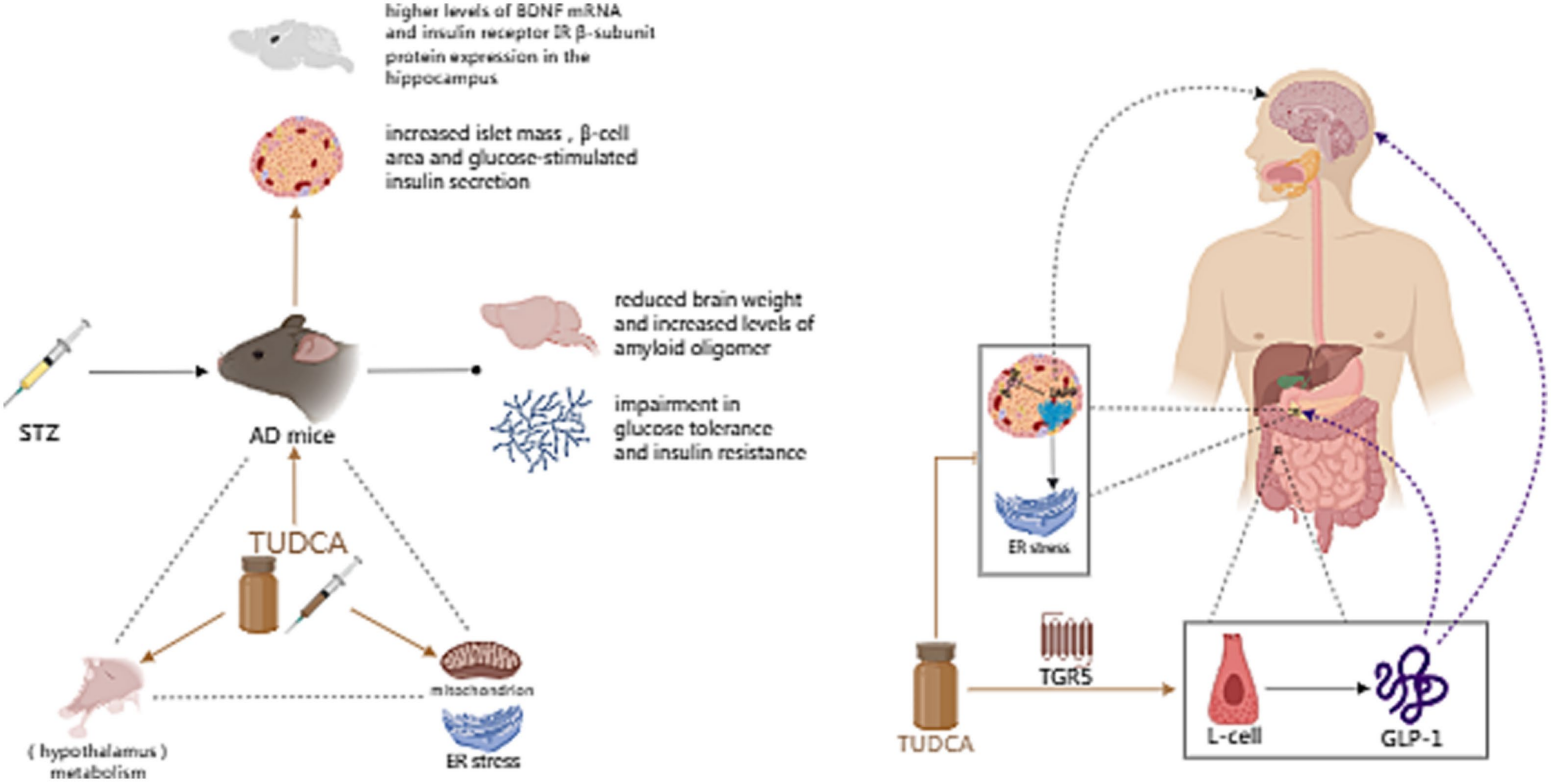
The effect of TUDCA in improving metabolic disorder. Metabolic disorders, such as diabetes and obesity, are closely associated with AD. Intracerebroventricular (ICV) injection of Streptozotocin (STZ), a chemical used to induce both type 1 and type 2 diabetes mellitus in rodents, can be used to create a mouse model of AD, which exhibits reduced brain weight, increased levels of amyloid oligomers, impaired glucose tolerance and insulin resistance. TUDCA treatment could increase islet mass, β-cell area and glucose-stimulated insulin secretion, as well as potentiate the neuroprotective effect of insulin. Furthermore, TUDCA treatment could restore the damaged hypothalamic leptin signaling pathway, alleviate peripheral ER stress and improve overall metabolism. The association between AD and metabolism is represented by the black dashed triangle, while the influence of TUDCA is highlighted by brown arrows. In addition, TUDCA has the potential to alleviate pathological changes induced by islet amyloid polypeptide (IAPP), while effectively stimulating the release of glucagon like peptide-1 (GLP-1) by activating the bile acid receptor TGR5 located on L-cells. The brown inhibitory arrow represents the potential of TUDCA to alleviate IAPP-induced pathological changes. Additionally, the TGR5 arrow, also depicted in brown, signifies the activation effect of TUDCA. GLP-1 can not only treat diabetes and obesity through a variety of mechanisms, such as stimulating insulin secretion, inhibiting glucagon secretion, protecting β cells and decreasing food intake, but also affect neurological and cognitive functions. The aforementioned functions of GLP-1 are illustrated by purple dashed arrows.

In another study conducted by Zangerolamo et al. (2021a), it was found that TUDCA treatment could restore the damaged hypothalamic leptin signaling pathway in STZ mice and mitigate energy metabolism disorders. Additionally, Ochiai et al. (2021) proposed that TUDCA may alleviate peripheral ER stress and improve metabolism, affecting the peripheral tissues and the brain. Consequently, this ameliorates the formation of amyloid pathology in the brain. The above studies indicate that TUDCA can impede the progression of AD pathogenesis by blocking the reciprocal advancement between AD and metabolic disorders (Figure 7).

## 5 Conclusion and future directions

Based on the evidence presented above, we conclude that there is a correlation between Alzheimer’s disease and the disorder of bile acid metabolism. Monitoring modifications happening in bile acid can serve as an early means of differentiating and screening for Alzheimer’s disease. Interventions targeting bile acids show potential for improving AD. Tauroursodeoxycholic acid (TUDCA), an endogenous bile acid, has certain therapeutic effects in AD.

The precise mechanism by which tauroursodeoxycholic acid functions is still not completely comprehended, including reducing Amyloid-β deposition, regulating apoptotic pathways, preventing hyperphosphorylation and aggregation of Tau, protecting neuronal synapses, anti-inflammatory effects, and improving metabolic disorders. At the same time, multiple signal pathways interact with each other and exhibit intricate interrelationships, necessitating additional elucidation. Issues such as whether TUDCA directly binds to species like Aβ/tau oligomers, and the physicochemical properties of TUDCA in the presence of different staged amyloid species also need to be addressed. Furthermore, conducting a comprehensive investigation into the association between AD and metabolic disorders, as well as the role of TUDCA in this relationship, could reveal a promising area of research for clarifying potential therapeutic targets. In addition, the current research findings predominantly revolve around animal experiments, and there exist discrepancies in the experimental outcomes, which could potentially be attributed to variations in the types of AD model mice utilized by researchers, as well as discrepancies in the methodologies employed during the research process.

In the future, the therapeutic effects of tauroursodeoxycholic acid need to be verified by more clinical studies, and the precise molecular pathways that are activated by TUDCA require further clarification to explore effective therapeutic targets for Alzheimer’s disease.

## Author contributions

HS: Writing – original draft. JLiu: Writing – original draft. LW: Writing – review & editing. XH: Writing – review & editing. JLi: Writing – review & editing. LZ: Writing – review & editing. RP: Writing – review & editing. AZ: Writing – review & editing.
